# Two new species of *Antrodia* (Polyporales, Basidiomycota) in western China

**DOI:** 10.3389/fmicb.2023.1102575

**Published:** 2023-02-13

**Authors:** Hong-Min Zhou, Shun Liu, Xiao-Juan Deng, Hong-Gao Liu, Rui Xing, Yu-Cheng Dai, Ying-Da Wu

**Affiliations:** ^1^Institute of Microbiology, School of Ecology and Nature Conservation, Beijing Forestry University, Beijing, China; ^2^College of Biological Science and Engineering, North Minzu University, Yinchuan, China; ^3^Faculty of Agronomy and Life Sciences, Zhaotong University, Zheaotong, China; ^4^Northwest Institute of Plateau Biology, Chinese Academy of Sciences, Xining, China; ^5^China Fire and Rescue Institute, Beijing, China

**Keywords:** fomitopsidaceae, multigene, taxonomy, wood-inhabiting fungi, phylogeny

## Abstract

Two new species of *Antrodia*, *A. aridula* and *A. variispora*, are described from western China. Phylogeny based on a six-gene dataset (ITS + nLSU + nSSU + mtSSU + TEF1 + RPB2) demonstrates that samples of the two species form two independent lineages within the clade of *Antrodia s.s.* and are different in morphology from the existing species of *Antrodia*. *Antrodia aridula* is characterized by its annual and resupinate basidiocarps with angular to irregular pores of 2–3 mm each and oblong ellipsoid to cylindrical basidiospores measuring 9–12 × 4.2–5.3 μm, growing on gymnosperm wood in a dry environment. *Antrodia variispora* is characterized by its annual and resupinate basidiocarps with sinuous or dentate pores with a size of 1–1.5 mm each and oblong ellipsoid, fusiform, pyriform, or cylindrical basidiospores measuring 11.5–16 × 4.5–5.5 μm, growing on the wood of *Picea*. The differences between the new species and morphologically similar species are discussed in this article.

## Introduction

*Antrodia* P. Karst. is one of the major groups of fungi causing brown rot, mostly on gymnosperm wood in the Northern Hemisphere (Gilbertson and Ryvarden, 1986; Núñez and Ryvarden, 2001; Ryvarden and Melo, 2017; Wu et al., 2022), and traditionally, the members of the genus are characterized by annual to perennial, leathery, mostly light colored, resupinate to effused-reflexed or distinctly pileate basidiocarps; a dimitic hyphal structure or a few species with a monomitic hyphal system; generative hyphae with clamp connections; skeletal hyphae negative in Melzer’s reagent; some species with amyloid skeletal hyphae; and hyaline, thin-walled basidiospores which are negative in Melzer’s reagent and cotton blue (Spirin et al., 2013; Liu et al., 2022). Recent studies demonstrated that this genus is polyphyletic including 12 small and monophyletic genera (Ortiz-Santana et al., 2013; Spirin et al., 2013; Runnel et al., 2019; Liu et al., 2022), and *Antrodia s. str.* Has been delimited as the species grouped around *Antrodia serpens* (Fr.) P. Karst. within the antrodia clade of Polyporales (Hibbett and Donoghue, 2001; Spirin et al., 2013).

During an investigation on polypores in western China, five resupinate, cream to buff specimens were collected from Qinghai Province and Inner Mongolia Autonomous Region, western China. Macromorphology and the ecology of brown rot on gymnosperm wood showed that they belong to *Antrodia s.l.*, and further morphological examination and phylogenetic analysis indicated that they represent two undescribed species of *Antrodia s. str*. Thus, we describe them as two new species in the present article.

## Materials and methods

### Morphological studies

All studied specimens are deposited in the herbarium of the Institute of Microbiology, Beijing Forestry University (BJFC). Morphological descriptions are based on field notes and microscopic examinations of voucher specimens. Special color terms are based on Anonymous (1969) and Petersen (1996). Microscopic structures refer to Spirin et al. (2013), Chen and Cui (2015), and Liu et al. (2022).

### DNA extraction, amplification, and sequencing

Acetyl trimethylammonium bromide rapid plant genome extraction kit (Aidlab Biotechnologies Co., Ltd., Beijing, China) was used to obtain DNA templates from dried specimens and perform the polymerase chain reaction (PCR) according to the manufacturer’s instructions with some modifications (Chen and Dai, 2021; Liu et al., 2022). The primers of ITS, including nLSU, nSSU, mtSSU, TEF1, and RPB2, for amplifying the DNA regions are mentioned in Table 1. The PCR procedure for ITS, mtSSU, TEF1, and RPB2 follows Liu et al. (2022). All newly generated sequences have been submitted to GenBank and are listed in Table 2.

**Table 1:** PCR primers used in this study.

Gene	Primer	Primer sequences (5′–3′)	References
ITS	ITS5	GGA AGT AAA AGT CGT AAC AAG G	White et al. (1990)
	ITS4	TCC TCC GCT TAT TGATAT GC	White et al. (1990)
nLSU	LR0R	ACC CGC TGA ACT TAA GC	Vilgalys and Hester (1990)
	LR7	TAC TAC CAC CAA GAT CT	Vilgalys and Hester (1990)
nSSU	MS1	CAG CAG TCA AGA ATATTA GTC AAT G	White et al. (1990)
	MS2	GCG GAT TAT CGA ATT AAATAA C	White et al. (1990)
mtSSU	MS1	CAG CAG TCA AGA ATATTA GTC AAT G	White et al. (1990)
	MS2	GCG GAT TAT CGA ATT AAATAA C	White et al. (1990)
TEF	983F	GCY CCY GGH CAY CGT GAY TTY AT	Rehner and Buckley (2005)
	1567R	ACH GTR CCR ATA CCA CCR ATC TT	Rehner and Buckley (2005)
RPB2	RPB2-5F	GAY GAY MGW GAT CAY TTY GG	Liu et al. (1999)
	RPB2-7cR	ATG GGY AAR CAA GCY ATG GG	Liu et al. (1999)

**Table 2:** Taxa information and GenBank accession numbers of the sequences used in this study.

Species name	Sample no.	Locality	ITS	nLSU	nSSU	mtSSU	TEF1	RPB2
*Anthoporia albobrunnea*	FP 100514	Unknown	EU232215	EU232299	EU232257	—	—	—
*Anthoporia albobrunnea*	S 4665	Russia	KY948808	KY948880	—	—	—	—
*Antrodia aridula*	Dai 24525	China	**OP854667**	**OP856750**	**OP856745**	**OP856741**	**OP851386**	**OP851381**
*Antrodia aridula*	Dai 24526	China	**OP854668**	**OP856751**	**OP856746**	**OP856742**	**OP851387**	**OP851382**
*Antrodia aridula*	Dai 24527	China	**OP854669**	**OP856752**	**OP856747**	**OP856743**	**OP851388**	**OP851383**
*Antrodia aridula*	Dai 24530	China	**OP854670**	**OP856753**	**OP856748**	**—**	**—**	**OP851384**
*Antrodia bambusicola*	Cui 11280	China	MG787579	MG787620	MG787726	MG787667	MG787845	MG787792
*Antrodia bambusicola*	Dai 11901	China	MG787580	MG787621	MG787727	MG787668	MG787846	MG787793
*Antrodia favescens*	JV 0309/103	USA	KC543127	MG787622	MG787729	MG787669	KC543178	MG787794
*Antrodia favescens*	JV 0412/4-J	USA	KC543129	KC543129	MG787730	MG787670	KC543179	MG787795
*Antrodia griseoflavescens*	Spirin 11175	Russia	MK119762	MK119762	—	—	—	—
*Antrodia griseoflavescens*	Kristiansen 2010	Norway	MK119763	MK119763	—	—	—	—
*Antrodia heteromorpha*	Dai 12755	USA	KP715306	KP715322	KR605908	KR606009	KP715336	KR610828
*Antrodia heteromorpha*	Dai 12742	USA	KP715319	ON417199	MG787728	MG787671	MG787847	KT895887
*Antrodia latebrosa*	Ryvarden 10031	Tanzania	MK119769	MK119769	—	—	—	—
*Antrodia macra*	Eriksson 1967	Unknown	KR605810	KR605749	KR605909	—	KR610739	MG787796
*Antrodia mappa*	RP 11756	Finland	KC543113	KC543113	—	—	—	—
*Antrodia mappa*	TN 2669	Canada	KC543130	KC543130	—	—	—	—
*Antrodia multiformis*	JV 1209/76	USA	KT381618	KT381618	—	—	—	—
*Antrodia multiformis*	JV 1307 9-J-1	USA	KT381619	KT381619	—	—	—	—
*Antrodia neotropica*	Cui 11141	China	MG787581	MG787623	—	MG787673	MG787848	MG787797
*Antrodia neotropica*	FLOR 54186	Brazil	KT970445	KT970454	—	—	—	—
*Antrodia parvula*	OM 18226	Indonesia	MK119764	MK119764	—	—	—	—
*Antrodia parvula*	OM 11589	Indonesia	MK119766	MK119766	—	—	—	—
*Antrodia peregrina*	Dai 3026	China	MK119767	MK119767	—	—	—	—
*Antrodia serpens*	Dai 7465	Luxemburg	KR605813	KR605752	KR605913	KR606013	KR610742	KR610832
*Antrodia serpens*	Dai 14850	Poland	MG787582	MG787624	MG787731	MG787674	MG787849	MG787798
*Antrodia subheteromorpha*	Cui 9617	China	MG787583	MG787625	MG787735	MG787675	MG787850	MG787799
*Antrodia subheteromorpha*	Cui 18416	China	MW377257	MW377338	MW377416	MW382052	MW337088	MW337025
*Antrodia subserpens*	Cui 8310	China	KP715310	KP715326	MG787732	MG787677	KP715340	KT895888
*Antrodia subserpens*	Dai 13233	China	KP715309	KP715325	MG787734	MH055437	KP715339	KT895889
*Antrodia tanakae*	Cui 9743	China	KR605814	KR605753	KR605914	KR606014	KR610743	KR610833
*Antrodia tanakae*	Dai 11770	China	KR605815	KR605754	KR605915	KR606015	KR610744	KR610834
*Antrodia tenerifensis*	Kout 13129	Spain	KY446066	KY446066	—	—	—	—
*Antrodia tenerifensis*	Kout 1412/2	Spain	KY446065	KY446065	—	—	—	—
*Antrodia variispora*	Dai 23995	China	**OP854671**	**OP856749**	**OP856744**	**—**	**—**	**OP851385**
*Brunneoporus kuzyana*	JV 0909/37	Czech Republic	KU866267	MG787628	MG787738	MG787680	KU866221	MG787803
*Brunneoporus kuzyana*	Spirin 6771	Russia	KU866265	MG787629	MG787739	MG787681	KU866218	MG787804
*Brunneoporus malicola*	Cui 7258	China	MG787586	MG787631	MG787741	MG787683	MG787853	MG787806
*Brunneoporus malicola*	Cui 7166	China	MG787585	MG787630	MG787740	MG787682	MG787852	MG787805
*Buglossoporus eucalypticola*	Dai 13660	China	KR605808	KR605747	KR605906	KR606007	KR610736	KR610825
*Buglossoporus eucalypticola*	Dai 13660A	China	KR605809	KR605748	KR605907	KR606008	KR610737	KR610826
*Buglossoporus quercinus*	JV 1406/1	Czech Republic	KR605801	KR605740	KR605899	KR606002	KR610730	KR610820
*Buglossoporus quercinus*	LY BR 2030	France	KR605799	KR605738	KR605897	KR606000	KR610728	KR610818
*Cartilosoma ramentacea*	Cui 16256	China	OK045506	OK045512	OK045494	OK045500	OK076960	OK076904
*Cartilosoma ramentacea*	Dai 6082	China	MG787595	MG787640	MG787750	MG787692	MG787860	MG787813
*Cartilosoma rene-hentic*	PRM 944766	Czech Republic	MK558725	—	—	—	—	—
*Daedalea modesta*	Cui 10151	China	KP171205	KP171227	KR605883	KR605986	KR610716	KR610806
*Daedalea modesta*	Cui 10124	China	KR605791	KR605730	KR605882	KR605985	KR610715	KR610805
*Daedalea quercina*	Dai 12152	Czech Republic	KP171207	KP171229	KR605886	KR605989	KR610717	KR610809
*Daedalea quercina*	Dai 12659	Finland	KP171208	KP171230	KR605887	KR605990	KR610719	KR610810
*Daedalella micropora*	Dai 18509	Malaysia	MW377286	MW377365	MW377444	MW382073	MW337113	MW337049
*Daedalella micropora*	E 7389	Indonesia	AJ542527	—	—	—	—	—
*Dentiporus albidoides*	X 1433	Italy	KC543147	KC543147	—	—	—	—
*Dentiporus albidoides*	X 1510	France	KC543168	—	—	—	—	—
*Flavidoporia pulverulenta*	Dai 15877	China	MG787588	—	MG787745	MG787687	MG787855	MG787810
*Flavidoporia pulvinascens*	Cui 10441	China	MG787590	MG787636	ON417019	MG787688	MG787857	MG787811
*Flavidoporia pulvinascens*	Cui 9542	China	MG787589	MG787635	MG787746	ON417078	MG787856	ON424764
*Fomitopsis betulina*	Cui 17121	China	OL621853	OL621242	OL621779	OL621753	OL588982	OL588969
*Fomitopsis betulina*	Dai 11449	China	KR605798	KR605737	KR605895	KR605998	KR610726	KR610816
*Fomitopsis hengduanensis*	Cui 16259	China	MN148232	OL621247	OL621782	OL621758	MN161747	MN158175
*Fomitopsis hengduanensis*	Cui 17056	China	MN148233	OL621248	OL621783	OL621759	MN161748	MN158176
*Fomitopsis pinicola*	AT Fp 1	Sweden	MK208852	—	—	—	MK236359	MK236362
*Fomitopsis pinicola*	HK 19330	Russia	KF169655	—	—	—	KF178380	KF169724
*Fragifomes niveomarginatus*	Cui 10108	China	KR605778	KR605717	KR605851	KR605955	KR610684	KR610776
*Fragifomes niveomarginatus*	Wei 5583	China	HQ693994	KC507175	KR605852	KR605956	KR610685	ON424771
*Neoantrodia angusta*	*VS* 6479	Russia	KT995127	KT995149	MG787756	MG787696	KU052718	MG787818
*Neoantrodia serialis*	JV 1509/5	Czech Republic	KT995120	KT995143	—	—	KU052726	—
*Neoantrodia serialis*	TP 243	Finland	KT995121	KT995144	—	—	KU052725	—
*Neolentiporus maculatissimus*	CIEFAP 93	Argentina	JX090122	—	—	—	—	—
*Neolentiporus maculatissimus*	CIEFAP 92	Argentina	JX090121	—	—	—	—	—
*Niveoporofomes spraguei*	JV 0509/62	USA	KR605786	KR605725	KR605864	KR605968	KR610697	KR610788
*Niveoporofomes spraguei*	4638	France	KR605784	KR605723	KR605862	KR605966	KR610696	KR610786
*Oligoporus rennyi*	Cui 17054	China	OK045508	OK045514	OK045496	OK045502	OK076962	OK076934
*Postia lacteal*	Cui 17334	China	OM039287	OM039187	OM039254	OM039222	OM037810	OM037782
*Pseudoantrodia monomitica*	Dai 13381	China	MG787602	ON417234	MG787768	—	MG787866	MG787822
*Pseudoantrodia monomitica*	Dai 10828	China	MG787601	MG787648	MG787767	—	MG787865	ON424803
*Pseudofomitopsis microcarpa*	Cui 16404	Vietnam	MW377316	MW377394	MW377473	MW382097	MW337139	—
*Pseudofomitopsis microcarpa*	Cui 16406	Vietnam	MW377317	MW377395	MW377474	MW382098	ON424865	—
*Rhizoporia hyalina*	*VS* 2532	Russia	JQ700267	JQ700267	—	—	—	—
*Rhizoporia hyalina*	Kotiranta-19668	Russia	JQ700284	JQ700284	—	—	—	—
*Rhodoantrodia tropica*	Dai 13428	China	MG787605	MG787652	MG787778	MG787708	MG787867	MG787823
*Rhodoantrodia tropica*	Dai 13434	China	MG817481	MG817479	MG787779	MG787709	—	MG787824
*Rhodoantrodia yunnanensis*	Han 1157	China	MT497886	MT497884	—	—	—	—
*Rhodoantrodia yunnanensis*	Zhao 4566	China	MT497887	MT497885	—	—	—	—
*Rhodofomes roseus*	Cui 10633	China	KR605782	KR605721	KR605860	KR605964	KR610693	KR610784
*Rhodofomes roseus*	JV 1110/9	Czech Republic	KR605783	KR605722	KR605861	KR605965	KR610694	KR610785
*Rhodofomes subfeei*	Cui 9229	China	KR605789	KR605728	KR605869	ON417098	KR610701	KR610793
*Rhodofomes subfeei*	Dai 10430	China	KR605788	KR605727	KR605868	KR605972	KR610702	KR610792
*Rhodofomitopsis feei*	Oinonen 6011906	Brazil	KC844851	KC844856	KR605837	KR605943	KR610671	KR610767
*Rhodofomitopsis pseudofeei*	Cui 16794	Australia	MK461952	MK461956	MK461964	MK461960	MK463986	MK463984
*Rhodofomitopsis pseudofeei*	Cui 16762	Australia	MK461951	MK461955	MK461963	MK461959	MK463985	MK463983
*Rubellofomes cystidiata*	Cui 5481	China	KF937288	KF937291	KR605832	KR605938	KR610667	KR610765
*Rubellofomes cystidiatus*	Yuan 6304	China	KR605769	KR605708	KR605833	KR605939	KR610668	—
*Subantrodia juniperina*	03010/1a	USA	MG787606	MG787653	MG787782	MG787712	MG787873	MG787831
*Subantrodia uzbekistanica*	Dai 17104	Uzbekistan	KX958182	KX958186	—	ON417103	ON424883	—
*Subantrodia uzbekistanica*	Dai 17105	Uzbekistan	KX958183	KX958187	—	ON417104	ON424884	—
*Ungulidaedalea fragilis*	Cui 10919	China	KF937286	KF937290	KR605840	KR605946	KR610674	KR610770

New sequences are shown in bold.

### Phylogenetic analysis

A total of 98 samples of *Antrodia* and related taxa were used for phylogenetic analysis in this study (Table 2). *Oligoporus rennyi* (Berk. & Broome) Donk and *Postia lactea* (Fr.) P. Karst. were selected as outgroups for phylogenetic analysis following Liu et al. (2022), based on the combined datasets of the internal transcribed spacer (ITS) region, the large subunit nuclear ribosomal RNA gene (nLSU), the small subunit nuclear ribosomal RNA gene (nSSU), the small subunit mitochondrial rRNA gene sequences (mtSSU), the translation elongation factor 1-α gene (TEF1), and the second subunit of RNA polymerase II (RPB2). Sequences were aligned with MAFFT v. 7 online^1^ adjusting the direction of nucleotide sequences according to the first sequence (accurate enough for most cases), selecting the G-INS-i iterative refinement method (Katoh et al., 2017). The aligned sequences were deposited at TreeBase (submission ID 29874).^2^

The analyses of maximum parsimony (MP), maximum likelihood (ML), and Bayesian inference (BI) refer to Liu et al. (2022).

## Results

### Phylogeny

The concatenated dataset included 98 ITS sequences, 90 nLSU sequences, 67 nSSU sequences, 64 mtSSU sequences, 70 TEF1 sequences, and 66 RPB2 sequences representing 54 taxa. There are 4,608 characters in the dataset, including 3,044 were constant, 144 were variable and parsimony-uninformative, and 1,420 were parsimony-informative. MP analysis yields a tree (TL = 8,189, CI = 0.323, RI = 0.700, RC = 0.226, HI = 0.677). “GTR + I + G” was the best model for the BI analysis, lset nst = 6, rates = invgamma; prset statefreqpr = dirichlet (1,1,1,1). The average standard deviation of split frequencies in the BI analysis was 0.008957. Branches that received bootstrap support for MP (MP-BS), ML (ML-BS), and BI (BPP) greater than or equal to 50% (MP-BS and ML-BS) and 0.95 (BPP) are considered as significantly supported, respectively.

**Figure 1:**
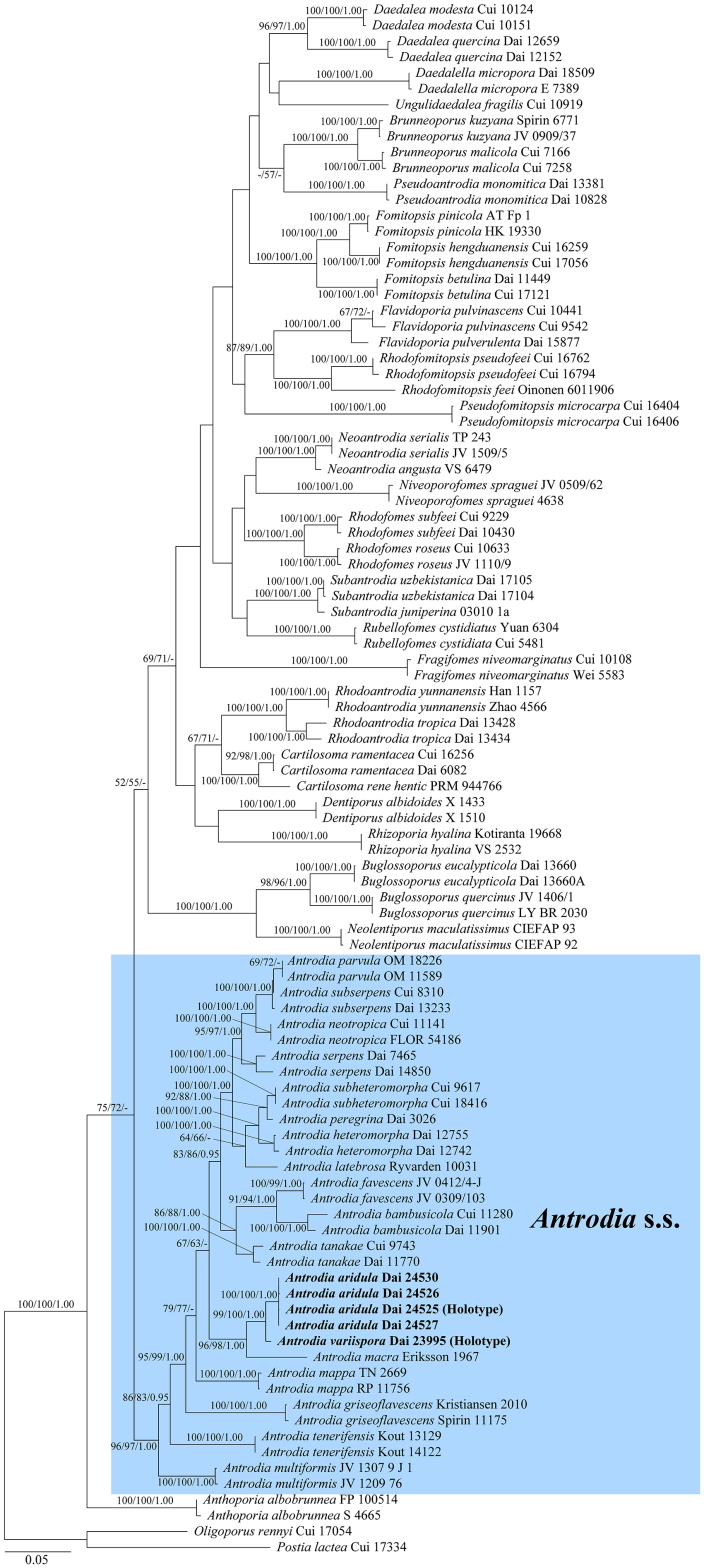
Maximum likelihood illustrating the phylogeny of *Antrodia* s.s. and related genera based on ITS + nLSU + nSSU + mtSSU + TEF1 + RPB2. Branches are labeled with parsimony bootstrap proportions and maximum likelihood bootstrap higher than 50%, and Bayesian posterior probabilities more than 0.95, respectively.

The current phylogeny placed all samples of *Antrodia* in a high supported clade (Figure 1). Two new species *Antrodia aridula* and *A. variispora* formed two well-supported lineages, respectively (100% MP 100% ML 1.00 BI and 99% MP 100% ML 1.00 BI). The two new species clustered with *Antrodia macra* (Sommerf.) Niemelä formed a well-supported subclade (96% MP 98% ML 1.00 BI).

### Taxonomy

**Figure 2:**
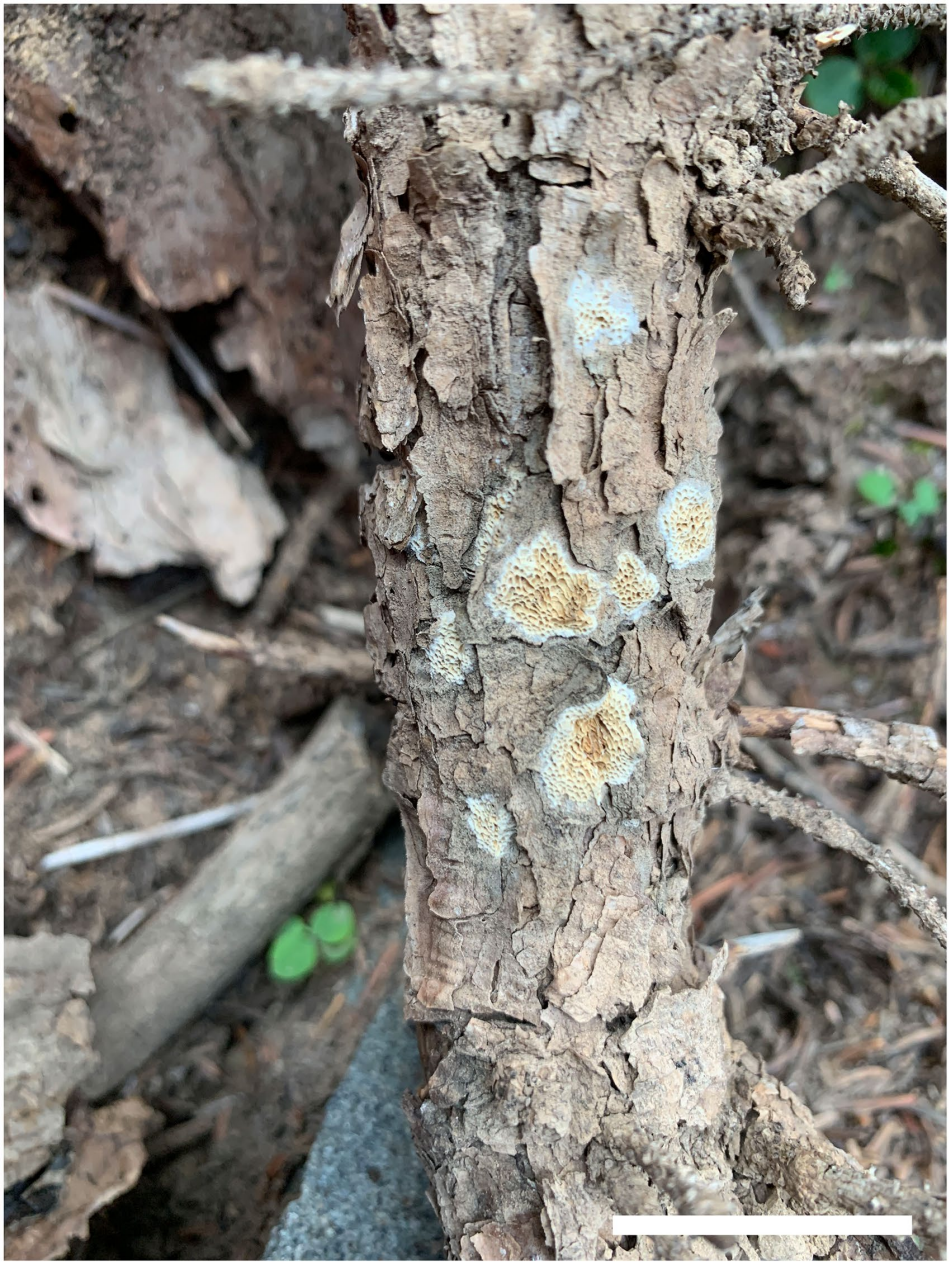
Basidiocarps of *Antrodia aridula* (holotype, Dai 24525). Scale bar: 2 cm.

**Figure 3:**
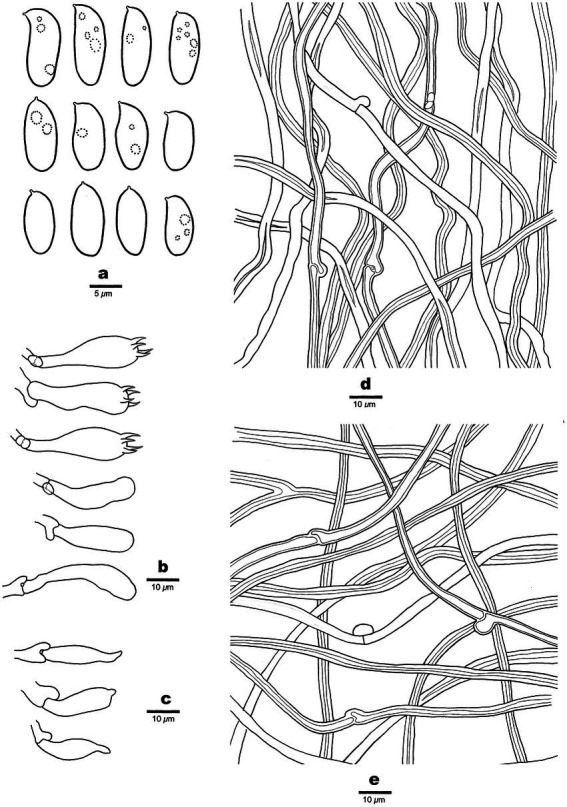
Microscopic structures of *Antrodia aridula* (holotype, Dai 24525). **(A)** Basidiospores. **(B)** Basidia and basidioles. **(C)** Cystidioles. **(D)** Hyphae from trama. **(E)** Hyphae from subiculum.

***Antrodia aridula*** Y.C. Dai, H.M. Zhou, Y.D. Wu & Shun Liu, sp. nov. Figures 2, 3.

MycoBank number: 846495.

Holotype: China. Inner Mongolia Autonomous Region, Alxa Left Banner, Helanshan Nature Reserve, elev. 2270 m, N 38.865282, E 105.899814, on fallen trunk of *Picea crassifolia*, 17 September 2022, Dai 24525 (BJFC).

Etymology: *Aridula* (Lat.): Referring to the species that prefer to the dry environment in Helan Mts. around the desert area of the Inner Mongolia Autonomous Region.

Basidiocarps: Annual, resupinate, tightly attached on wood, leathery when fresh, hard corky when dry, up to 5 cm long, 2 cm wide, and 0.5-mm thick at the center. Pore surface cream when fresh, becoming cream to buff when dry; sterile margin thinning out, white, up to 1 mm wide; pores angular to irregular, 2–3 per mm; dissepiments thin, lacerate. Subiculum cream, hard corky, paler contrast with tubes, up to 0.1-mm thick. Tubes concolorous with pores, hard corky, up to 0.4-mm long.

Hyphal structure: Hyphal system dimitic; generative hyphae with clamp connections; skeletal hyphae IKI–, CB–; tissue unchanged in KOH.

Subiculum: Generative hyphae hyaline, thin-to slightly thick-walled, rarely branched, 2–3 μm in diam; skeletal hyphae dominant, hyaline, thick-walled with a narrow lumen to subsolid, rarely branched, 2–5 μm in diam.

Tubes: Generative hyphae frequent, hyaline, thin-to slightly thick-walled, rarely branched, 2–3 μm in diam; skeletal hyphae dominant, hyaline, thick-walled with a narrow lumen to subsolid, rarely branched, interwoven, 3–4 μm in diam. Cystidia absent; cystidioles present, fusoid, thin-walled, 21–26 × 5–7 μm. Basidia clavate, with a basal clamp connection and four sterigmata, 23–30 × 9–10 μm; basidioles in shape similar to basidia, but smaller.

Spores: Basidiospores oblong ellipsoid to cylindrical, gently arcuate and tapering toward the apiculus, hyaline, thin-walled, smooth, sometimes within a few small guttules, IKI–, CB–, (8.5–)9–12(−12.8) × (4–)4.2–5.3(−5.6) μm, L = 10.31 μm, W = 4.7 μm, Q = 2.14–2.22 (*n* = 90/3).

Additional specimens examined (paratypes): China. Inner Mongolia Autonomous Region, Alxa Left Banner, Helanshan Nature Reserve, elev. 2270 m, N 38.865282, E 105.899814, on fallen trunk of *Picea crassifolia*, 17 September 2022, Dai 24526 (BJFC), Dai 24527 (BJFC); Helanshan Forest Park, elev. 2070 m, N 38.971847, E 105.905048, on fallen branch of *Pinus tubaeliformis*, 18 September 2022, Dai 24530 (BJFC).

Notes: *Antrodia aridula* is characterized by its annual and resupinate basidiocarps with angular to irregular pores of 2–3 per mm, a dimitic hyphal structure, the presence of fusoid cystidioles, basidiospores oblong ellipsoid to cylindrical, gently arcuate and tapering toward the apiculus, 9–12 × 4.2–5.3 μm, and growing on gymnosperm wood in a dry environment of West China. *Antrodia aridula* and *Cartilosoma ramentaceum* (Berk. & Broome) Teixeira (≡ *Antrodia ramentacea* (Berk. & Broome)) Donk have similar pores and growing on the wood of *Pinus*, but the latter differs from the former by gelatinous basidiocarps, smaller basidiospores (6.2–8.1 × 2.1–2.7 μm vs. 9–12 × 4.2–5.3 μm) and wider geographical distribution (Niemelä, 2005). *Cartilosoma* is different from *Antrodia* by completely resupinate and soft basidiocarps when fresh, and gelatinous hymenophore (Liu et al., 2022). *Antrodia aridula* is similar to *A. macra* (Sommerf.) Niemelä in macromorphology, and both species are closely related (Figure 1), but the latter species has smaller basidiospores (7.4–11 × 3–4.3 μm vs. 9–12 × 4.2–5.3 μm, Niemelä, 2005), and growing on *Salix* and *Populus* only (Ryvarden and Melo, 2017). *Antrodia aridula* is also closely related to *Antrodia variispora* (Figure 1), but the latter species has bigger and variable basidiospores (9–12 × 4.2–5.3 μm vs. 11.5–16 × 4.5–5.5 μm).

Ecologically, *Antrodia aridula* grows on fresh fallen gymnosperm trunks and branches in a dry environment, indicating a pioneer decayer in the coniferous forests of western China.

**Figure 4:**
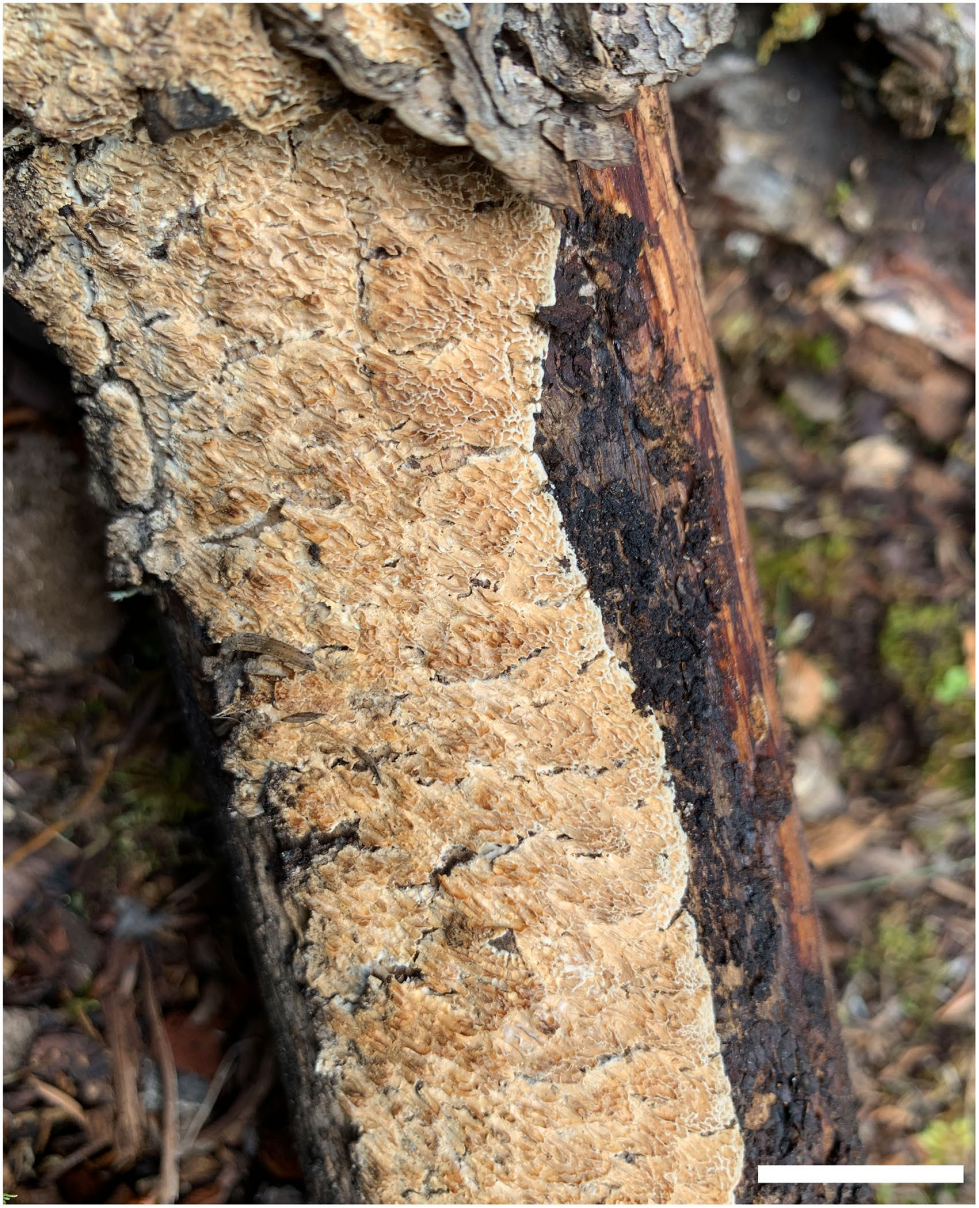
Basidiocarp of *Antrodia variispora* (holotype, Dai 23995). Scale bar: 2 cm.

**Figure 5:**
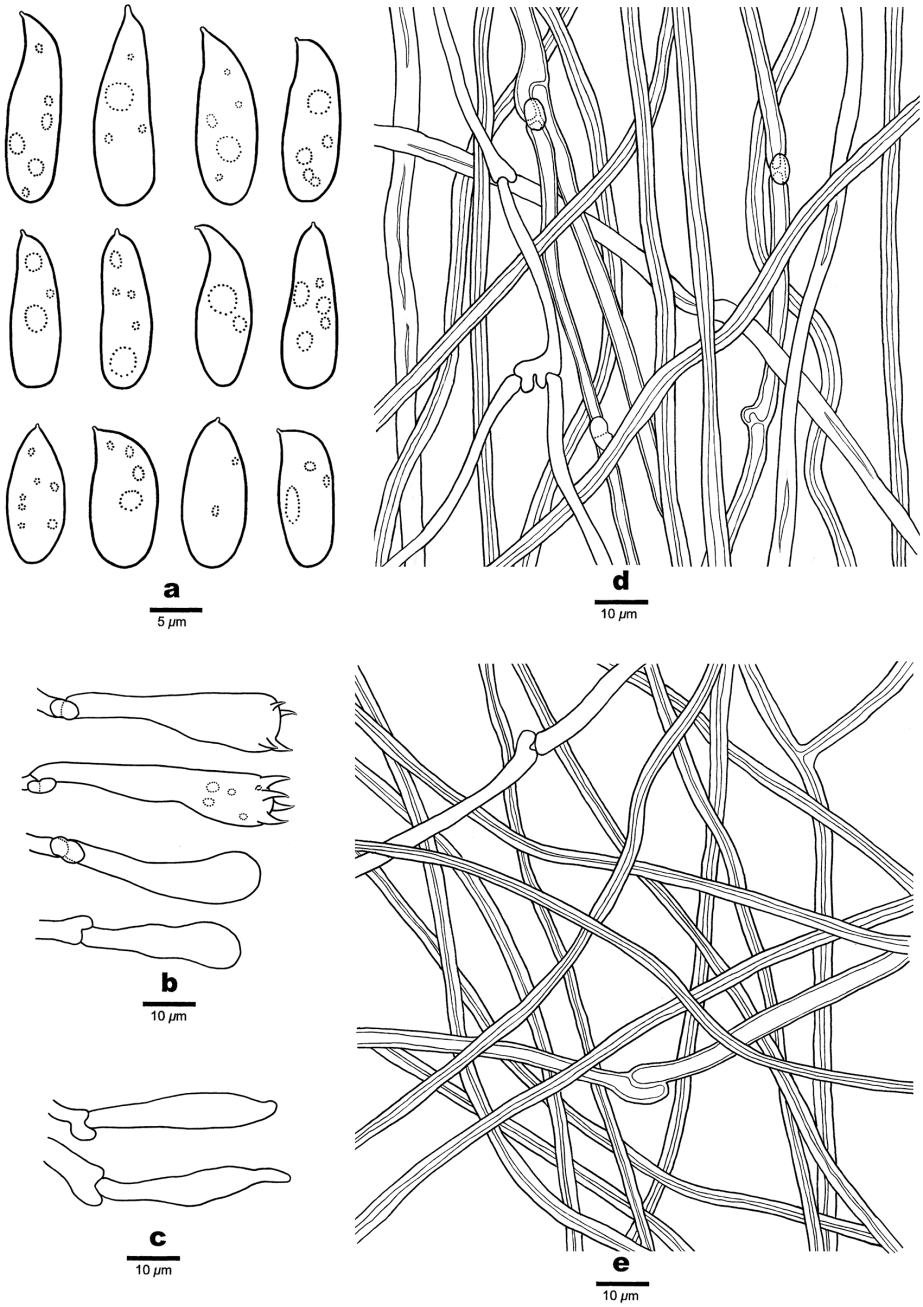
Microscopic structures of *Antrodia variispora* (holotype, Dai 23995). **(A)** Basidiospores. **(B)** Basidia and basidioles. **(C)** Cystidioles. **(D)** Hyphae from trama. **(E)** Hyphae from subiculum.

***Antrodia variispora*** Y.C. Dai, H.M. Zhou, Y.D. Wu & Shun Liu, sp. nov. Figures 4, 5.

MycoBank number: 846496.

Holotype: China. Qinghai Province, Nangqian County, Baizha Forest Park, elev. 4090 m, N 31.855040, E 96.467402, on stump of *Picea likiangensis* var. *balfouriana*, 7 August 2022, Dai 23995 (BJFC).

Etymology: *Variispora* (Lat.): Referring to the species having variable basidiospores.

Basidiocarp: Annual, resupinate, tightly attached to wood, leathery when fresh, hard corky to rigid when dry, up to 16 cm long, 6 cm wide, and 1.3-mm thick at the center. Pore surface cream when fresh, becoming cinnamon-buff when dry; sterile margin very narrow to almost lacking; pores sinuous or dentate, (0.5–)1–1.5(−2) per mm; dissepiments thin, lacerate. Subiculum cream, hard corky, paler contrast with tubes, up to 0.3-mm thick. Tubes concolorous with pores, rigid, up to 1-mm long.

Hyphal structure: Hyphal system dimitic; generative hyphae with clamp connections; skeletal hyphae IKI–, CB–; tissue unchanged in KOH.

Subiculum: Generative hyphae infrequent, hyaline, thin-to slightly thick-walled, rarely branched, 2–4 μm in diam; skeletal hyphae dominant, hyaline, thick-walled with a narrow lumen to subsolid, rarely branched, interwoven, 2.5–4 μm in diam.

Tubes: Generative hyphae frequent, hyaline, thin-to slightly thick-walled, frequently branched, 2.5–4 μm in diam; skeletal hyphae dominant, hyaline, thick-walled with a narrow lumen to subsolid, occasionally branched, interwoven, 3–5 μm in diam. Cystidia absent; cystidioles present, fusoid, thin-walled, 22–41 × 4.5–5 μm. Basidia clavate to pyriform, with a basal clamp connection and four sterigmata, 24–35 × 7–10 μm; basidioles in shape similar to basidia, but smaller.

Spores: Basidiospores variable, oblong ellipsoid, fusiform, pyriform or cylindrical, gently arcuate and tapering toward the apiculus, hyaline, thin-walled, smooth, usually within a few small guttules, IKI–, CB–, (11–)11.5–16(−18.5) × 4.5–5.5(−5.8) μm, L = 13.47 μm, W = 5.12 μm, Q = 2.63 (*n* = 30/1).

Notes: *Antrodia variispora* is characterized annual and resupinate basidiocarps with sinuous or dentate pores of 1–1.5 per mm, a dimitic hyphal structure, the presence of fusoid cystidioles, basidiospores variable, oblong ellipsoid, fusiform, pyriform or cylindrical, 11.5–16 × 4.5–5.5 μm, and growing on the wood of *Picea* in West China. *Antrodia variispora* is similar to *Adustoporia sinuosa* (Fr.) Audet (≡ *Antrodia sinuosa* (Fr.) P. Karst.) in macromorphology, but the latter species has distinctly smaller basidia (11–15 × 4–5 μm vs. 24–35 × 7–10 μm, Ryvarden and Gilbertson, 1993) and smaller basidiospores (4.9–6 × 1.4–1.8 μm vs. 11.5–16 × 4.5–5.5 μm, Niemelä, 2005). *Adustoporia* differs from *Antrodia* s. str. by pale brown pore surface when fresh, smaller basidia (15–22 × 4–5 μm), smaller basidiospores (4–6 × 1–2 μm, Audet, 2017b). *Antrodia variispora* and *Dentiporus albidoides* (A. David & Dequatre) Audet (≡ *Antrodia albidoides* A. David & Dequatre) have similar pore and basidiospore dimensions (Ryvarden and Melo, 2017), but the latter species has skeletocystidia, uniformed cylindrical basidiospores, and growing on angiosperm wood in Mediterranean area (Ryvarden and Melo, 2017). In addition, phylogenetically both species are distantly related (Figure 1). The genus *Dentiporus* differs from *Antrodia* s. str by often resupinate basidiocarps usually with rose pink tinted pore surface and round to irpicoid pores (Audet, 2017a). *Antrodia variispora* is closely related to *A. macra* (Figure 1), but *A. macra* has regularly cylindrical to oblong ellipsoid basidiospores which are smaller than those in *Antrodia variispora* (7.4–11 × 3–4.3 μm vs. 11.5–16 × 4.5–5.5 μm, Niemelä, 2005).

Ecologically, *Antrodia variispora* grows on a large stump of *Picea likiangensis* var. *balfouriana* in a virgin forest, and we tried to find more samples in a similar environment of the same forest, and unfortunately, we did not find the second sample. Thus, *Antrodia variispora* seems to be a rare species in old growth forests.

## Discussion

Adding the two new species from China, the definition of *Antrodia* s. str. is modified as follows: Basidiocarps annual, resupinate to effused-reflexed, soft corky to leathery when fresh, become corky to hard corky or rigid when dry; pileal surface glabrous or matted, white, cream to brownish gray; pore surface white to cream when fresh, buff to pale brown upon drying; pores round, angular, sinuous or dentate; subiculum or context cream, corky; hyphal system dimitic with clamped generative hyphae; skeletal hyphae IKI−, CB−; cystidia absent; fusoid cystidioles present or absent; basidiospores long (6.5–16 μm), oblong ellipsoid, cylindrical, fusiform or pyriform, hyaline, thin-walled, smooth, IKI−, CB−; causing a brown rot.

## Data availability statement

The datasets presented in this study can be found in online repositories. The names of the repository/repositories and accession number(s) can be found in the article.

## Author contributions

Y-CD coordinated the project and designed the experimental plan. H-MZ, Y-CD, and SL analyzed the data. Y-CD, X-JD, and H-GL collected the samples from the field. H-MZ and Y-CD wrote the original draft. H-MZ, Y-CD, Y-DW, and SL reviewed and edited the manuscript. Y-CD acquired funding. All authors contributed to the article and approved the submitted version.

## Funding

The research was supported by the National Natural Science Foundation of China (Project Nos. 32161143013, 31870007). We are grateful to Zhan-Bo Liu was a companion on the field trips.

## Conflict of interest

The authors declare that the research was conducted in the absence of any commercial or financial relationships that could be construed as a potential conflict of interest.

## Publisher’s note

All claims expressed in this article are solely those of the authors and do not necessarily represent those of their affiliated organizations, or those of the publisher, the editors and the reviewers. Any product that may be evaluated in this article, or claim that may be made by its manufacturer, is not guaranteed or endorsed by the publisher.

## Footnotes

^1^ https://mafft.cbrc.jp/alignment/server/

^2^ http://purl.org/phylo/treebase
